# Biomechanical, ultrastructural, and electrophysiological characterization of the non-human primate experimental glaucoma model

**DOI:** 10.1038/s41598-017-14720-2

**Published:** 2017-10-30

**Authors:** VijayKrishna Raghunathan, J. Seth Eaton, Brian J. Christian, Joshua T. Morgan, James N. Ver Hoeve, Chen-Yuan Charlie Yang, Haiyan Gong, Carol A. Rasmussen, Paul E. Miller, Paul Russell, T. Michael Nork, Christopher J. Murphy

**Affiliations:** 1 Department of Surgical and Radiological Sciences, School of Veterinary Medicine, University of California – Davis, Davis, California, 95616 United States of America; 2 Ocular Services On Demand (OSOD), Madison, Wisconsin 53719 United States of America; 3 The Ocular Surface Institute, Department of Basic Sciences, College of Optometry, University of Houston, Houston, Texas 77204 United States of America; 4 Covance Laboratories, Inc., Madison, Wisconsin 53704 United States of America; 5 Department of Ophthalmology and Visual Sciences, School of Medicine and Public Health, University of Wisconsin – Madison, Madison, Wisconsin 53792 United States of America; 6 Department of Anatomy and Neurobiology, School of Medicine, Boston University, Boston, Massachusetts 02118 United States of America; 7 Department of Ophthalmology, School of Medicine, Boston University, Boston, Massachusetts 02118 United States of America; 8 Department of Surgical Sciences, School of Veterinary Medicine, University of Wisconsin – Madison, Madison, Wisconsin 53706 United States of America; 9 Department of Ophthalmology & Vision Science, School of Medicine, University of California – Davis, Sacramento, California, 95817 United States of America

## Abstract

Laser-induced experimental glaucoma (ExGl) in non-human primates (NHPs) is a common animal model for ocular drug development. While many features of human hypertensive glaucoma are replicated in this model, structural and functional changes in the unlasered portions of trabecular meshwork (TM) of laser-treated primate eyes are understudied. We studied NHPs with ExGl of several years duration. As expected, ExGl eyes exhibited selective reductions of the retinal nerve fiber layer that correlate with electrophysiologic measures documenting a link between morphologic and elctrophysiologic endpoints. Softening of unlasered TM in ExGl eyes compared to untreated controls was observed. The degree of TM softening was consistent, regardless of pre-mortem clinical findings including severity of IOP elevation, retinal nerve fiber layer thinning, or electrodiagnostic findings. Importantly, this softening is contrary to TM stiffening reported in glaucomatous human eyes. Furthermore, microscopic analysis of unlasered TM from eyes with ExGl demonstrated TM thinning with collapse of Schlemm’s canal; and proteomic analysis confirmed downregulation of metabolic and structural proteins. These data demonstrate unexpected and compensatory changes involving the TM in the NHP model of ExGl. The data suggest that compensatory mechanisms exist in normal animals and respond to elevated IOP through softening of the meshwork to increase outflow.

## Introduction

Glaucoma is a blinding disease of the retina and optic nerve, projected to affect over 70 million people worldwide by the year 2020^1^. Retinal ganglion cells (RGC) are particularly sensitive to elevation in intraocular pressure (IOP), a common clinical feature of many forms of human glaucoma. In response, RGC bodies and their axons that comprise the retinal nerve fiber layer, (RNFL) undergo irreversible degeneration leading to loss of visual function. Long-term glaucoma therapy often requires topical medications to reduce IOP, the majority of which act at the eye’s anterior segment to reduce aqueous humor production or enhance its outflow^2–5^.

The structure of the eye’s aqueous humor outflow system and its influence on IOP have been studied in humans and animals for many years and continue to be investigated. Of the available animal models, experimental glaucoma (ExGl) in the non-human primate (NHP), induced by subtotal laser photocoagulation of the trabecular meshwork (TM), is considered the most predictive for drug efficacy in the human^6^. Morphological and hydrodynamic data in this model suggest that fibrosis of the TM and adjacent inner wall of Schlemm’s canal (SC) reduces the area for conventional aqueous outflow, leading to decreased outflow facility, and elevated IOP^7–11^. Furthermore, the classic arcuate mid-peripheral visual field losses observed in human patients with primary open angle glaucoma (POAG) and elevated IOP have also been observed in visual field testing of NHPs with ExGl^12^. Additional evidence suggests that aqueous humor flow in eyes with ExGl is largely diverted to the small unlasered area of TM, suggesting a capacity for this tissue to dynamically compensate both structurally and functionally, accommodating the increased flow^9^. Compensation has not, however, been directly demonstrated in this model.

It remains unclear whether tissue changes in the TM of human eyes with glaucoma represent primary structural abnormalities that lead to elevation of IOP, or develop as a consequence of chronically elevated IOP. Using atomic force microscopy (AFM), our group has previously shown that the matrix around the TM’s juxtacanalicular (JCT) region (adjacent to the inner walls of SC), is, on average, 20 times stiffer in tissue from glaucomatous human eyes compared to that of non-glaucomatous donor eyes^13^. However, due to a history of chronically elevated IOP in the available glaucomatous donors, we were unable to identify whether TM stiffening was the primary event or a consequence of elevated IOP. If TM stiffening was a consequence of the latter, it would be anticipated that chronic elevation in IOP in the experimental primate model would result in similarly increased stiffness of the matrix.

To investigate this hypothesis, we obtained whole globes or corneoscleral buttons containing TM from cynomolgus monkeys (*Macaca fascicularis*) with chronic unilateral laser trabeculoplasty-induced ExGl. All animals had been previously used to screen topical hypotensive agents for efficacy in the context of glaucoma drug development programs. Animals were euthanized for reasons unrelated to the study herein. Establishment and progression of ExGl had been thoroughly documented in these animals using a variety of electrodiagnostic and advanced ocular imaging approaches. In the context of these data, and to better understand and define structural changes of the anterior segment in this model, we analyzed the normal (non-lasered) regions of TM using AFM, proteomics, and confocal, light, and transmission electron microscopy (TEM).

## Results

Compared to normal left eyes, all right eyes with ExGl maintained higher IOPs following laser trabeculoplasty for a minimum of 40.1 months (median 69.5 months; range 40.1–137.7 months) (Table 1). IOP values presented for right eyes with ExGl represent the mean ± SD for measurements taken for approximately 1 year prior to euthanasia. IOP values presented for untreated left eyes are those taken immediately prior of euthanasia.

**Table 1:** Summary of intraocular pressure (IOP) measurements, retinal nerve fiber layer thicknesses, and other procedures and analyses performed prior to and following euthanasia.

	**Animal ID**	**IOP (mmHg) Prior to Euthanasia**		**Retinal Nerve Fiber Layer Thickness (µm) Measured Using** ***In Vivo*** **SD-OCT**			**ERG/VEP**	**Atomic Force Microscopy (AFM)**	**Proteomic Analysis**	**Transmission Electron Microscopy (TEM)**
		**Mean ± SD OD (Right Eye)** ††	**OS (Left Eye)** §	**OD (Right Eye)**	**OS (Left Eye)**	**% Difference between OD and OS**				
Set A* (N = 3)	21	35.8 ± 4.4	18.0	57	109	47.71				✓
	34	36.6 ± 2.4	19.3	47	105	55.24				✓
	32	24.1 ± 2.8	22.0	100	112	10.71				✓
Set B** (N = 6)	1	23.0 ± 1.4	18.0	29	106	72.64	✓	✓		
	2	43.4 ± 8.7	14.7	47	104	54.81	✓	✓		
	3	45.5** ± **9.9	21.2	43	94	54.26	✓	✓		
	4	48.5 ± 7.9	25.8	29	91	68.13	✓	✓		
	5	46.9 ± 11.5	22.7	32	106	69.81	✓	✓		
	6	56.4 ± 9.4	25.2	33	101	67.33	✓	✓		
Set C† (N = 7)	28	26.8 ± 1.7	14.3	99	104	4.81		✓	✓	
	36	24.8 ± 1.5	20.7	104	109	4.59		✓	✓	
	19	29.5 ± 1.4	29.0	95	100	5.00			✓	
	24	37.4 ± 4.1	23.0	41	92	55.43			✓	
	10	30.8 ± 2.9	16.3	86	96	10.42			✓	
	18	24.3 ± 1.6	15.3	85	89	4.49			✓	
	20	24.0 ± 1.4	22.7	94	98	4.08			✓	

^*^Whole globes submitted for confocal, light, and transmission electron microscopy.

^**^Corneoscleral rims submitted for proteomic analysis and/or atomic force microscopy.

^†^Corneoscleral rims submitted for proteomic analysis only.

^††^Mean IOP ± SD OD from measurements taken over approximately 1 year prior to euthanasia.

^§^IOP measurement OS immediately prior to euthanasia.

### SD-OCT and Retinal Nerve Fiber Layer Thickness (RNFLT)

**Figure 1:**
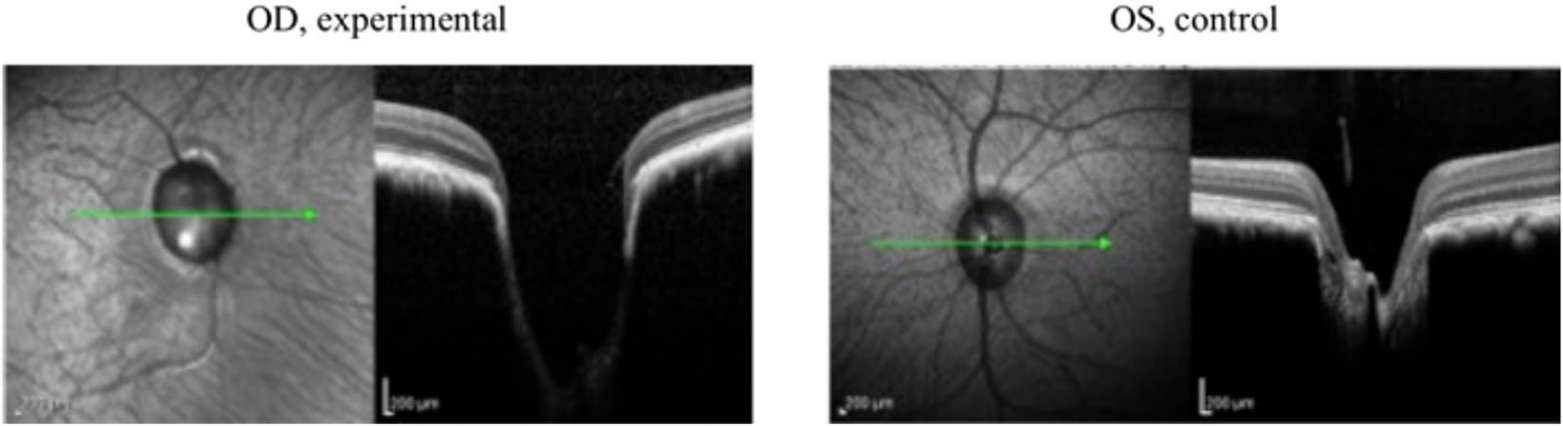
Representative pre-euthanasia OCT images from Animal Number 5 demonstrate optic nerve head (ONH) cupping, neuroretinal rim thinning, and posterior displacement of the lamina cribrosa. (OD = Right Eye; OS = Left Eye).

In eyes with ExGl, SD-OCT images from Set A and C animals demonstrated cupping of the ONH, neuroretinal rim thinning, and posterior displacement of the lamina cribrosa (see Fig. 1). Most Set B animals showed mild to moderate cupping. In Set A animals, both Cirrus™ and Spectralis™ instruments confirmed significant loss of RNFLT, and to comparable degrees, at each interval. Likewise, RNFLT measurements from each instrument, determined using both automated and manual segmentation algorithms, detected comparable loss of RNFL. As expected, most eyes with ExGl had reduced RNFLT compared to normal control eyes. Remarkably, however, considerable RNFLT thinning was not found in all eyes with ExGl (see Table 1).

### Electrodiagnostics

#### ERG and VEP in ExGl

**Figure 2:**
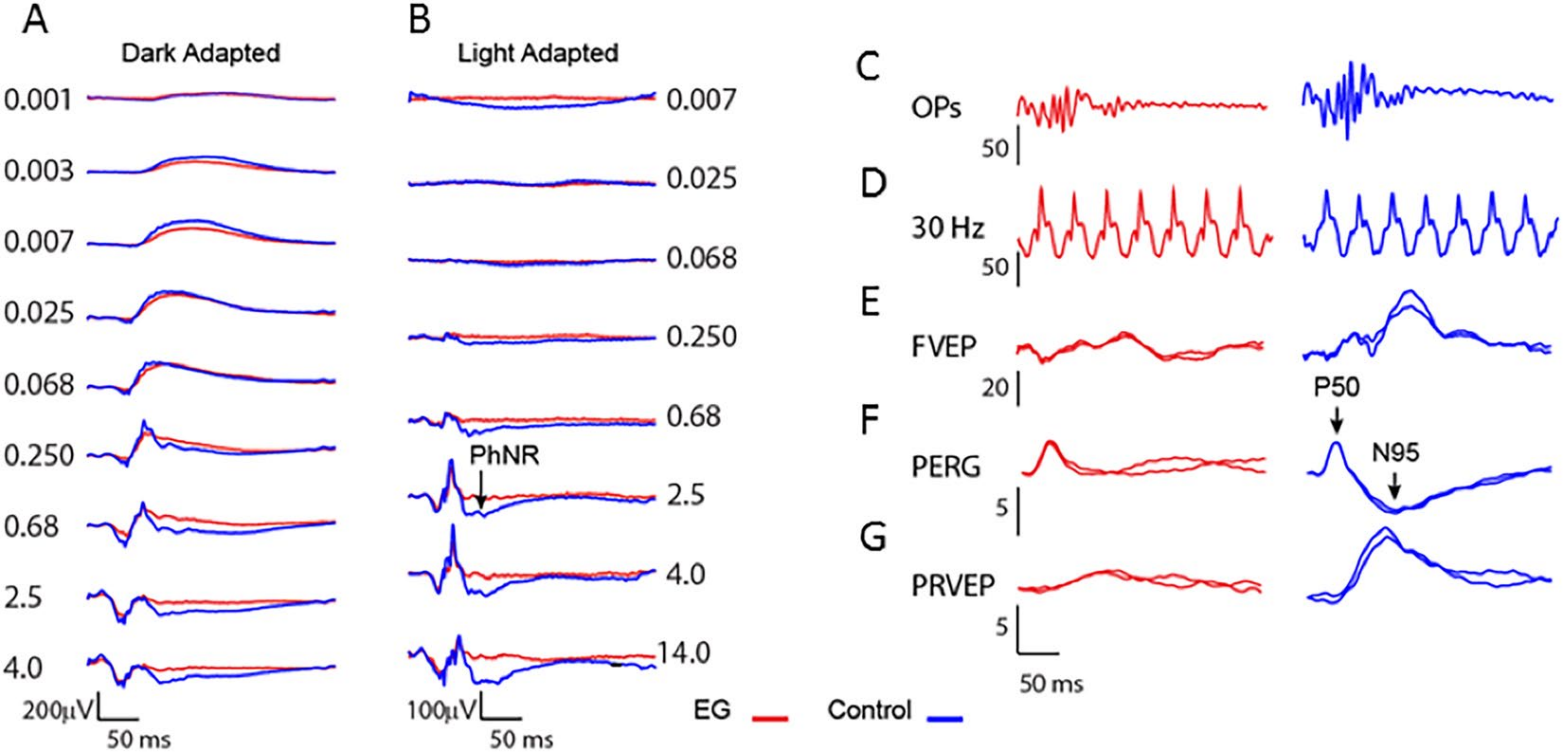
**Examples of electrophysiologic recordings from Set B (n = 6 animals). ExGl eye indicated in red, fellow control eye in blue**. (**A**) Dark-adapted full-field ERG flash intensity series. (**B**) Light adapted full-field single flash intensity series. Note the emergence of the photopic negative response (PhNR) at the higher flash strengths. (**C**) Oscillatory potentials (OPs) band-pass filtered from the DA 2.5 flash intensity ERG. **(D**) Light adapted full-field 30 Hz flicker. **(E**) Light adapted flash-evoked cortical potential (FVEP). (**F**) ERG evoked by checkerboard pattern reversing at 2 Hz (PERG). (**G**) cortical VEP evoked by the 2 Hz pattern stimulus.

**Figure 3:**
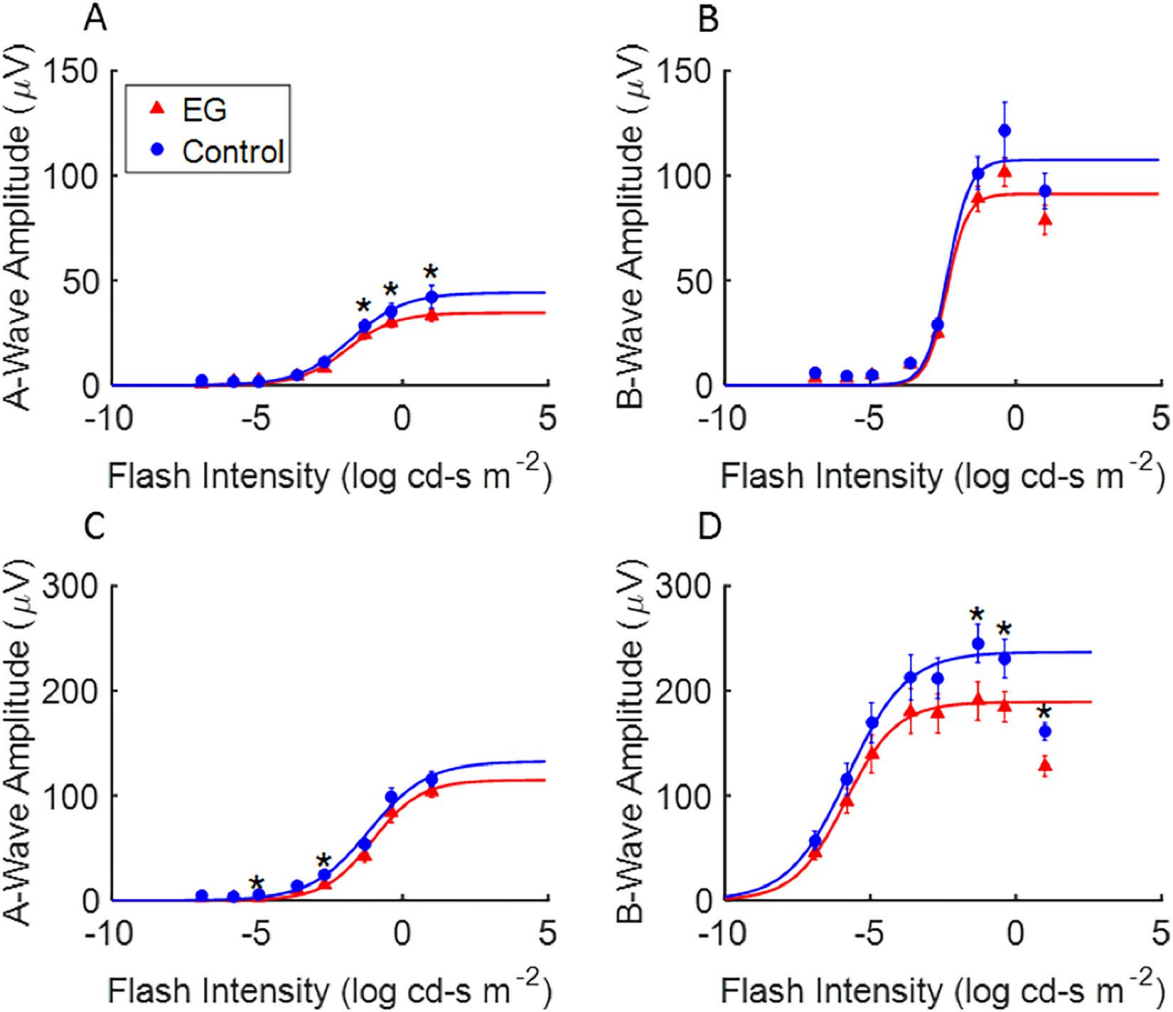
Flash intensity series under dark- and light- adapted conditions for ExGl eyes (Red, triangles) and fellow control eyes (Blue, circles) in Set B (n = 6 animals). (**A**) Dark adapted a-wave amplitude versus flash strength. (**B**) Dark adapted b-wave amplitude versus flash strength. (**C**) Light adapted a-wave amplitude versus flash strength, (**D**) Light adapted b-wave amplitude versus flash strength. Data points fitted with compressive non-linear function (see text).

Certain features of the ERGs and VEPs elicited by both full-field flash stimulation and transient pattern reversal stimulation differed between ExGl and fellow control eyes. Figure 2 shows representative traces from dark adapted and light adapted flash intensity series, OPs, FVEP to full-field flash stimulation, and PERGs and PRVEPs to 2 Hz checkerboard stimulation. Full-field scotopic and photopic ERGs and A- and B-wave ERGs were similar between ExGl and control eyes at low and moderate flash strengths. However, ExGl amplitudes became relatively depressed in eyes with ExGl at high flash strengths (Fig. 3 **, asterisks)**. The data were fit to the hyperbolic non-linear function,

$$V=\frac{{V}_{max}\bullet {I}^{n}}{{I}^{n}+{\sigma }^{n}}$$

where V_max_ is the maximal response, I = flash intensity, n = sensitivity exponent, and σ, the half-saturation constant. The fitted curves show that V_max_ is consistently lower in ExGl eyes whereas the threshold and gain are similar between the eyes (see figure caption for details).

**Figure 4:**
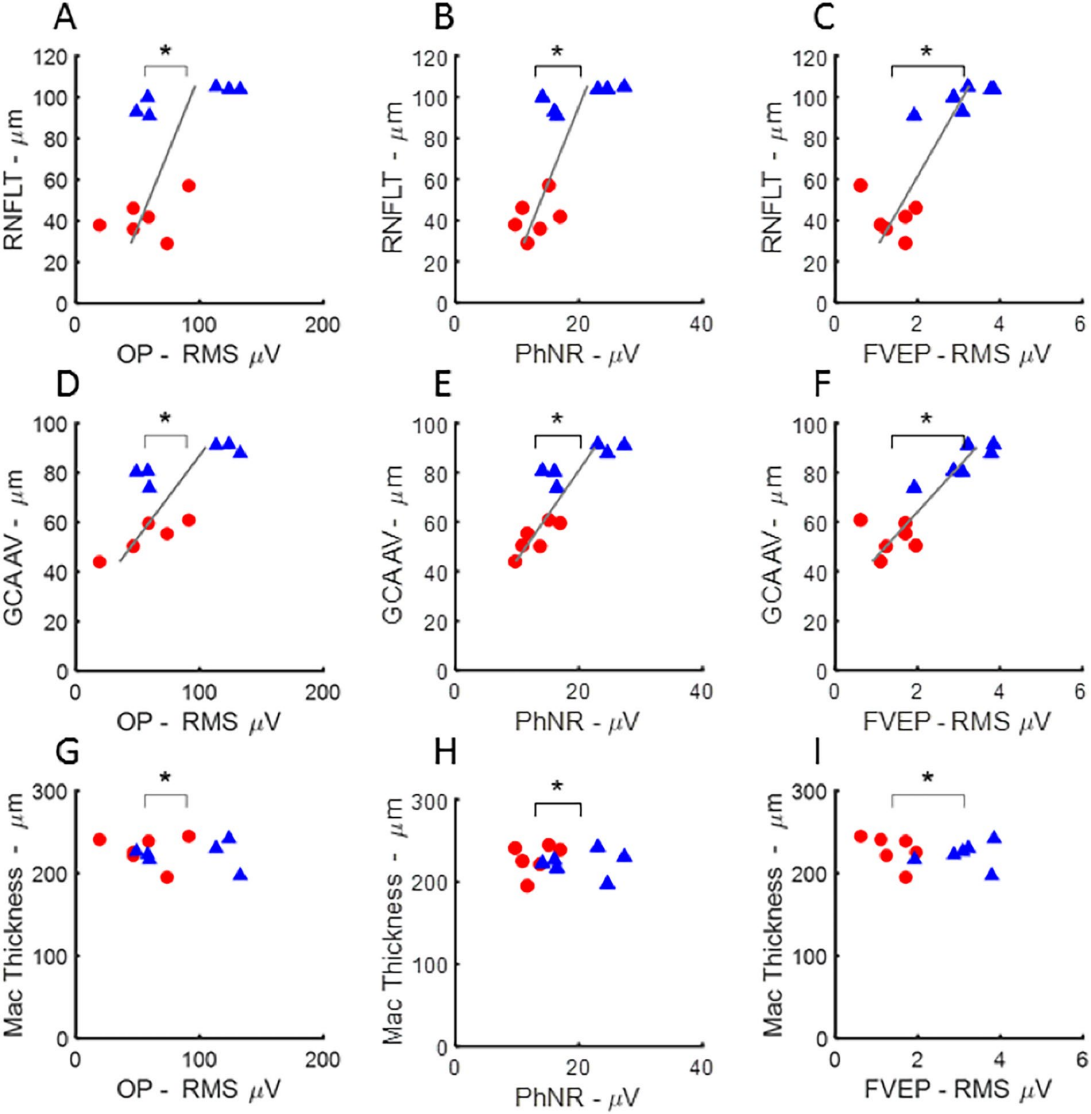
Scatter plots illustrating mean differences and correlations between full-field electrophysiological measures and OCT measures of retinal nerve fiber layer thickness (RNFLT from manual segmentation of Spectralis™), average ganglion cell analysis from the Cirrus™ (GCA AV), and macular thickness estimates from the Cirrus™ (Mac Thickness) in Set B (n = −6 animals). Full-field electrophysiology parameters plotted versus OCT measures for ExGl (red, circles) and control (blue, triangles) eyes. Bracket tick marks indicate the means of the parameters for ExGl and control eyes. Significant differences between eyes for the parameter are indicated by asterisks. Significantly thinner segmented OCT layers were present in ExGl eyes, compared with controls, for RNFLT (p < 0.01) and GCA AV (p < 0.01), but not macular thickness (p > 0.6). Significantly lower amplitude electrophysiologic responses were present in ExGl eyes for full-field electrophysiology measures OPs (p < 0.01), PhNRs (p < 0.01), and FVEPs (p < 0.01). Significant correlations between RNFLT electrophysiology parameters are indicated by the presence of a gray line showing the least-squares linear fit. Significant correlations between RNFLT and the full-field electrophysiology measures were found for PhNR (p < 0.01), OPs (p < 0.01) and FVEP (p < 0.01), **A**–**C**). GCA AV also showed strong positive correlations with OP, PhNR and FVEP amplitudes, **D**–**E**) Macular Thickness was not affected by ExGl and was not correlated with these full-field electrophysiology measures **G**–**I**). Additional plots of full-field ERGs and OCTs are shown in the Supplemental Data.

**Figure 5:**
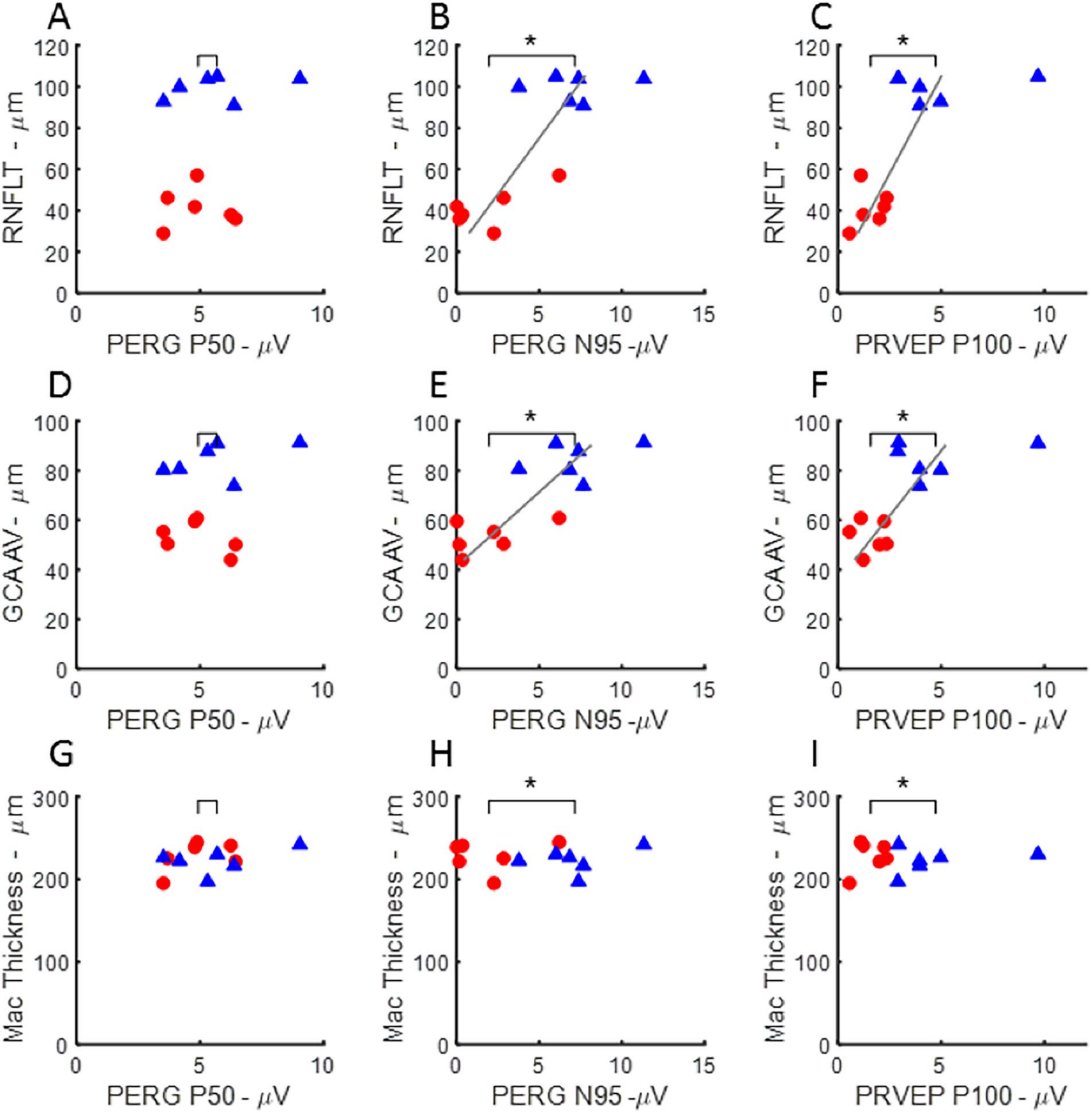
Relationship between electrophysiology elicited by pattern-stimulation and OCT measure in eyes with ExGl (red, circles) and fellow control eyes (blue, triangles) from Set B (n = 6 animals). Significant differences between means and significant correlations indicated as in Fig. 4. PERG N95 and PRVEP P100, but not PERG P50 amplitudes were significantly lower in ExGl compared with fellow control eyes, indicated by starred horizontal brackets (]*). RNFLT was strongly correlated with PERG N95 and PRVEP P100, but not PERG P50, **A–C)**. Likewise, PERG N95 and PRVEP P100 amplitudes were highly correlated with GCA AV, but not the PERG P50 (**D–F**). There was no correlation between Macular Thickness OCT and pattern-elicited ERG or VEP measures (**G–I**).

SD-OCT parameters including RNFLT, the Cirrus™ macular ganglion cell analysis (GCA, average) and macular thickness were examined for their relationship with electrophysiological functional measures. In Fig. 4, a star above the brackets in each graph indicates a significant difference (p < 0.05) in electrophysiology for the indicated parameter in full-field testing between eyes with ExGl and untreated control eyes. The location of the mean for the ExGl and control electrophysiology measures is indicated by downward ticks of the brackets. In all graphs, RNFLT and GCA differed significantly between ExGl and fellow control eyes; however, macular thickness did not differ. Figure 4 also shows that eyes with ExGl had consistently lower OPs, PhNRs, and FVEPs compared to control eyes. These features of the full-field ERG are thought to reflect function of second and higher-order retinal neurons and are affected in human and experimental glaucoma^14–16^. Figure 5 shows a similar plot for pattern stimulation. As indicated by the starred brackets, PERG N95 and PRVEP P100 were significantly depressed in ExGl eyes. As expected, the PERG P50 wave, considered to reflect the macular retina relatively independent of retinal ganglion cells, was not affected by ExGl^17,18^. In contrast, the PERG N95 wave, which has been linked with retinal ganglion cell and optic nerve function in many studies, was significantly reduced in ExGl eyes compared with the fellow eye (Fig. 5). The PRVEP ‘P100’-like wave was also depressed in ExGl eyes, as would be expected based on anatomy and many studies in humans with glaucoma^19,20^.

#### Correlation Between Electrodiagnostic Measurements and RNFLT

Figures 4 and 5 show the relationship between SD-OCT measures and ExGl and fellow control eyes. A significant nonparametric correlation is indicated by a least-squares line. Note that there are 4 possible outcomes for each plot: ExGl and control eyes could differ 1) only in an OCT parameter and not differ in electrophysiology, 2) only in electrophysiology and not in OCT, 3) in both OCT and electrophysiology with no correlation between the two, and 4) outcome 3 plus a correlation between the anatomical measure and the functional measure. Figure 4 shows that certain electrophysiology measures from full-field ERG fall into outcome 4, namely OPs, PhNR and FVEPs, differ in ExGl and control eyes, which also differ in RNFLT and GCA but not macular thickness, and that these electrophysiology measures are positively correlated with RNFLT and GCA, but not with macular thickness. Similarly, Fig. 5, shows that the PERG N95 and PRVEP P100 wave were significantly lower in ExGl and that these measures correlate significantly with RNFLT and GCA, but not with macular thickness. Note that the PERG P50 wave is not correlated with RNFLT or GCA, as would be expected given the association with macular retinal response independent of ganglion cell function. Fig. S1 shows that the full-field A- and B-waves recorded under light- and dark-adapted conditions, while differing between ExGl and Control eyes, show little relationship to the SD-OCT measures.

In summary, the results of a battery of non-invasive visual electrophysiologic measures presents a pattern that is consistent with the literature on these measures in human and experimental glaucoma. Full-field ERG responses were similar between ExGl and fellow eyes at low and moderate flash strengths; certain features of the full-field ERG, including the OPs and PhNR, which are ERG measures thought to depend on inner retinal function, were depressed in ExGl; pattern stimulation resulted in large differences between ExGl and fellow eyes. These functional measures are also correlated with RNFLT and the GCA. Of note, these measures were obtained using the Heidelberg Spectralis ™_ and Zeiss Cirrus™ instruments, the former measuring optic nerve head RNFL and the latter measuring ganglion cell and RNFL thickness around the macula (the agreement between the two machines measures of RNFLT was high; only the Spectralis™ RNFLT data are presented). There is very good agreement between the estimates of ganglion cell density and RNFLT, using different instruments, and their correlation with functional measures. In contrast, the thickness of the central macula, relatively devoid of ganglion cells, was not correlated with the perturbation in electrophysiology measures resulting from ExGl.

### Atomic Force Microscopy (AFM)

**Figure 6:**
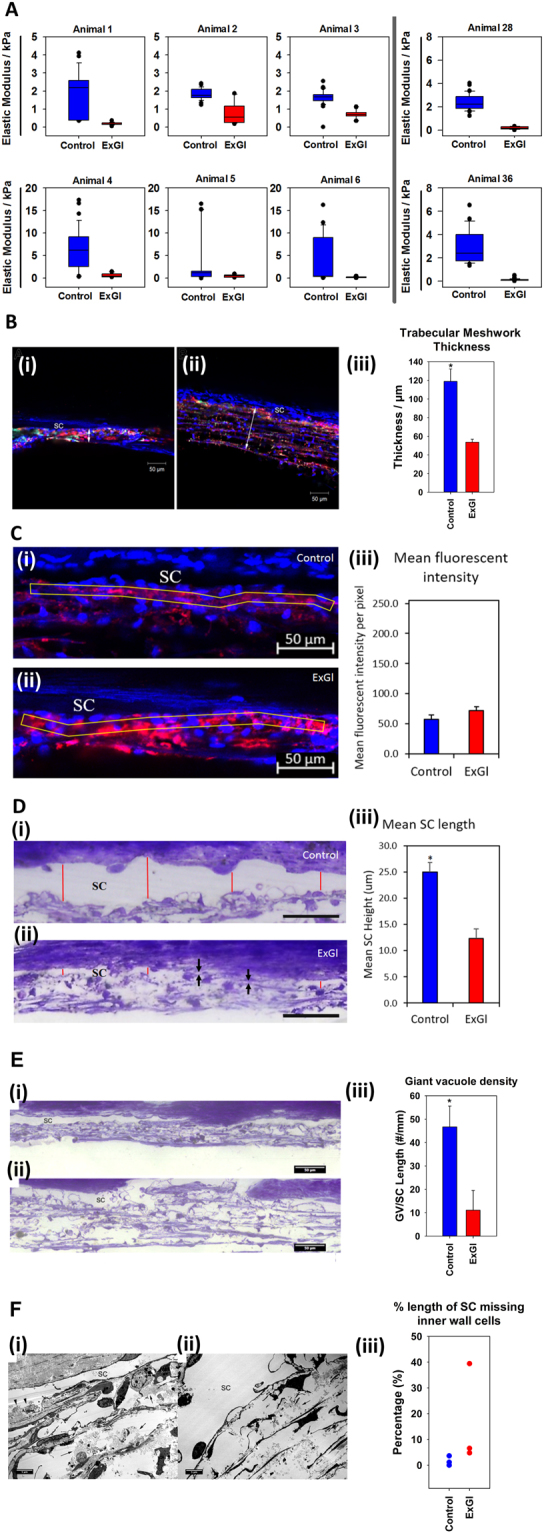
Comparison of results of atomic force microscopy (AFM), confocal microscopy, light microscopy, and transmission electron microscopy (TEM) between eyes with experimental glaucoma (ExGl/OD) and untreated control (OS) eyes (n = 8 animals for AFM; n = 3 animals for microscopy). (**A**) Box and whisker plots demonstrating the relative elastic moduli of untreated normal eyes (blue) and eyes with ExGl (red). (**B**) Comparison of the trabecular meshwork (TM) thickness between eyes with ExGl and normal control eyes. *(i-ii)*: Representative confocal images show that the TM thickness (double arrow) was smaller in a high-tracer region of an eye with ExGl (i) compared to similar region of a normal eye (ii). SC = Schlemm’s canal. *(iii)*: The TM thickness was significantly smaller in the high-tracer regions of eyes with ExGl compared to similar regions of the normal eyes. *: p < 0.05. Fluorescence of the tracer dye is indicated in red. (**C**) Fluorescence Intensity *(i-ii)* Representative confocal images showed no significant difference in *(iii)* fluorescence intensity between non-lasered regions of ExGl eyes and similar region of normal control eyes. (**D**) Height of Schlemm’s canal (SC). *(i-ii)*: Representative light microcopy images showed that the *(iii)* height of SC was significantly narrower in ExGl than normal control eyes (*red lines*). SC was collapse in the some regions (*arrows*) in ExGl eyes. (**E**): Giant vacuole density. *(i-ii)*: Representative light microcopy images showed significantly fewer giant vacuoles in the non-lasered region of an eye with ExGl (i) compared to the high-tracer region of a normal eye (ii). *(iii)*: Significantly more giant vacuoles were found in the high-tracer region of normal eyes compared to the non-lasered region of eyes with ExGl (*p < 0.05). (**F**) Comparison of ultrastructure of the Schlemm’s canal (SC) and juxtacanalicular tissue (JCT) between eyes with ExGl and normal control eyes. *(i)*: A representative electron microscopy image of the JCT and inner wall of SC from an eye with ExGl. Loose and expanded JCT was seen in the high-tracer region (arrow). SC was narrower compared to normal control eye, some inner wall endothelial cells along SC were lost (arrowheads), and some tracers were observed inside SC. More tracers were observed in the high-tracer regions of the eyes with ExGl compared to normal eyes. *(ii)*: An electron microscopy image of the JCT and inner wall region from a normal eye. Loose and expanded JCT was seen in the high-tracer region (arrow). SC was open and inner wall cells were intact. *(iii)*: Percent length of SC missing inner wall endothelial cells. More inner wall endothelial cells along SC appeared to be lost in the eyes with ExGl compared to the normal eyes.

We investigated if there were any correlation between elevated IOP and TM stiffness. The elastic moduli of the TM from both eyes of 6 animals (Set B) are shown in Fig. 6A. Elastic moduli were also determined for 2 animals from Set C. The mean elastic moduli of the control TM was 3.31 ± 0.32 kPa for Set B and 2.63 ± 0.14 kPa for two animals from Set C. Mean elastic moduli for the unlasered regions of TM in all eyes with ExGl were approximately 6-fold lower (0.464 ± 0.036 kPa for Set B, and 0.151 ± 0.014 kPa for Set C), indicating marked softening of the TM. Unexpectedly, the moduli values from the unlasered TM of eyes with ExGl were grouped around 300 Pa (i.e. 0.30 kPa), regardless of the difference in IOP between the eyes of the individual animals (Fig. S2). Even in the two Set C animals with only a small relative difference in the thickness of the RNFL, the elastic moduli of the TM were consistent with the values of the other monkeys measured.

### Proteomic Analysis

A total of seven of the unlasered regions of TM samples obtained from eyes with ExGl (Set C) were directly utilized for proteomic analysis. Among these, TM samples from two monkeys with only small differences in the RNFL ratios (animals #28 and #36) were analyzed for proteomics after modulus measurement. Principal component (PC) analysis was performed on the normalized spectral data of proteins comparing the ExGl group with control group. Scree plot demonstrated that 65% of the variance in the data was accounted by PCs 1 and 2 (Fig. S3). Clustering observed in the plot of PC1 versus PC2 demonstrated that significant differences in protein expression between groups existed (Fig. S3).

Approximately 1500 proteins were identified, and 11 proteins showed a significant difference (p < 0.05) in samples from normal and glaucomatous eyes, and are listed in Table 2. These 11 proteins include collagen VI (α3), as well as the matrix proteins osteoglycan, fibulin-1, NESH, and tenascin. Myocilin, a matricellular protein that is upregulated in TM after glucocorticoid treatment, was also found to be decreased. The exact functional role of myocilin in TM pathophysiology remains poorly understood but mutations in this gene have been shown to result in glaucoma^21–24^. A second group of proteins identified by the analyses was decreased in 5 of the samples and were close to or below the level of detection in the remaining samples. In this group of 23 proteins, several are associated with matrix (e.g. fibromodulin and complement factor H) while many are typically thought to be internal to cells. Since our samples were from fresh tissues, both TM cells and some inner walls of Schlemm’s canal (SC) would be present in our samples. Curiously, several enzymes were from the anabolic hexose monophosphate shunt, suggesting that this key metabolic pathway is down-regulated. Finally, a third group of 11 proteins was identified in 4 or 5 of the samples with the remaining samples below the level of detection. These proteins include collagen 1 (α2), collagen IV (α-1, 2, 4, and 5), heparin sulfate, proteoglycan, and laminin α5. All of these were significantly decreased in the samples from eyes with ExGl relative to the normal controls.

**Table 2:** Proteins decreased at least 2-fold in the unlasered trabecular meshwork (TM) of monkey eyes with experimental glaucoma (ExGl).

Group 1 - Decreased In All Samples	Group 2 - Decreased In 5/7 Samples (Undetected In Others)	Group 3 - Decreased In 4/7 Samples except #11; Not detected in #7 or #8)
Collagen α3 (VI)^71^	Fibromodulin^72,73^	Collagen α2 (I)
Prelamin A/C	Gelsolin^74^	Collagen α1 (IV)
Histone H2B^75^	Serotransferrin^76^	Collagen α(4 (IV)
Alpha enolase^77,78^	Glyceraldehyde-3-phosphate dehydrogenase	Basement membrane-specific heparin sulfate proteoglycan
Myocilin^79^	Fructose bisphosphate aldolase A	Smooth muscle actin
Osteoglycan^80^	Transketolase	Emulin
Fibulin-1^81^	Phosphoglycerate kinase-1	Laminin α-5
Tenascin^82^	Immunoglobulin ƛ	Fibulin-5
Nesh-SH3^83^	Retinal dehydrogenase	Collagen α5 (IV)
	Immunoglobulin γ	Collagen α2 (IV)
	α-2-macroglobulin^84^	Aldehyde dehydrogenase
	Immunoglobulin α	
	Elongation factor 1-α	
	Triosephosphate isomerase^78^	
	Sushi repeat protein SRPX2^85^	
	Procollagen C-endopeptidase enhancer 1^86^	
	Serpine peptidase inhibitor^87^	
	Dihydropyrimidinase related protein 3	
	Destrin	
	Cofilin-1	
	Apolipoprotein E^88^	
	Complement factor H^89^	
	Phosphoglycerate mutase^78^	

### Morphological Changes

A total of 67 microscopic images (a minimum of 8 images per eye) were analyzed from 3 pairs of eyes of 3 animals (Set A) to evaluate ultrastructural changes to the TM, and the inner wall of SC and the JCT.

#### Trabecular meshwork thickness

TM thickness, measured in confocal images, was found to be significantly less in the high-tracer regions of eyes with ExGl (i.e. unlasered region of ExGl eyes) compared to similar regions of the normal eyes (mean 53.7 ± 3.1 vs. 127.4 ± 24.0 μm, p = 0.03) (Fig. 6B).

#### Fluorescence Intensity Measurement

While there was visually a trend towards greater amounts of tracers in the high-flow regions of the eyes with ExGl compared to normal eyes by examining the confocal microscopy images; this difference was not statistically significant as quantified by fluorescence intensity measurement (71.6 ± 6.3 in ExGL vs 57.4 ± 8.9 in normal eyes, p = 0.2 (Fig. 6C).

#### Height of SC

The height of SC was significantly narrower or collapsed in some regions in eyes with ExGl compared to normal eyes. Mean SC height (the distance between the inner and outer walls of SC) was 12.3 ± 2.2 µm in ExGl compared to 25.0 ± 1.8 µm in normal eyes, *p < *0.001 (Fig. 6D **)**.

#### Giant vacuole density

To determine the giant vacuole (GV) density in light microscopic images, the length of inner wall of SC was analyzed ranged from 0.92–1.73 mm (the length being small due to only 1 clock-hour of tissue segment being left non-lasered in the eyes with ExGl). Significantly more giant vacuoles were found in the high-tracer regions of the normal eyes compared to those with high tracer regions with ExGl (mean 47 ± 9 vs 11 ± 8 GVs per mm SC, p < 0.01) (Fig. 6E).

#### Ultrastructure of the inner wall of Schlemm’s canal (SC) and juxtacanalicular tissue (JCT)

By TEM, similar morphology was observed in the high-tracer regions of the JCT of both normal eyes and those with ExGl (i.e corresponding to unlasered region of ExGl eyes), where the JCT was loose and expanded. More inner wall endothelial cells of SC appeared to be lost in the eyes with ExGl compared to the normal eyes (Fig. 6F).

## Discussion

Most forms of glaucoma are associated with elevated IOP, believed to develop as a result of progressive reduction in aqueous outflow facility. In the animals in this study, sustained increase in IOP was successfully established by inducing subtotal fibrosis of the TM, effectively reducing outflow area. In these animals, *in vivo* OCT of the fundus confirmed the presence of characteristic features of ExGl including cupping of the ONH, neuroretinal rim thinning, posterior displacement of the lamina cribrosa, and decreased RNFL thickness, indicative of RGC loss^25–28^. As with humans, NHPs with advanced stages of glaucoma tend to exhibit a “floor effect” in SD-OCT derived measurements. With this phenomenon, RNFL and ganglion cell-inner plexiform layer (GCIPL) thickness values level off, with RNFLT rarely falling below 35 µm, and almost never below 25 µm in NHPs, possibly due to the presence of residual glial or non-neural tissue (including blood vessels and displaced amacrine cells). Correspondingly, some electrodiagnostic abnormalities were expected in eyes with chronic ExGl and matched well with published data in humans with glaucoma^18,29^, while others were unexpected. Notably, there were strong correlations between OPs, PhNR, FVEP, PERG N95, P100 and RNFLT and GCA, but not with macular thickness. Other functional measures, including scotopic and photopic full-field ERG single-flash a- and b-waves did not demonstrate a corresponding correlation (see supplemental figures). Selective reduction of the ERG waveforms elicited only at high intensity levels argues against optic nerve injury alone being the sole retinal damage caused by glaucoma^30–33^. It is of interest that in ExGl eyes, there was a relatively reduced full-field ERG A- and B-wave amplitude at the highest flash intensities. It is unlikely that this is the consequence of ischemic processes following elevation of IOP, since the response to dim flashes was not affected and responses are not correlated with RNFLT thinning. Furthermore, it suggests that optic nerve injury alone cannot account for the results, since optic nerve transection does not lower ERG amplitudes^15,34^. One possibility that has been suggested by Nork *et al*., is that there may be a process in ExGl initiated by transient ischemia of cone photoreceptors that is relatively independent of a direct IOP-induced ganglion cell death pathway^33^. This could result in a reduced response to bright flashes that recruit cones in the dark-adapted eye as well as responses to strong flashes that also contain a contribution from cone photocurrent.

It is noteworthy that among the animals included in this study, there was a range of RNFL thinning, as previously reported with optic nerve axon counts in NHPs with ExGl^33^. In some eyes with ExGl, including two that eventually underwent AFM, RNFL thinning was minimal compared to control eyes, indicating mitigated damage to RGCs, despite documented elevation in IOP. These data are consistent with previous reports showing that some animals and human patients are more resistant to loss of RGCs even with high IOPs^25,27,33^, and may be analogous to human patients with clinically normal visual fields despite ocular hypertension^35,36^. Interestingly, AFM in the same two animals mentioned above demonstrated a decrease in stiffness in the JCT area in the unlasered region of the eye with ExGl similar to that of the first six animals that were measured. Thus, these data strongly suggest that any relative neuroprotection in these eyes is likely mediated by a mechanism independent of the compensatory softening of the TM and JCT.

The consistent and marked softening of the unlasered portion of the TM in eyes with chronic ExGl represents a biomechanical alteration, to our knowledge, previously undocumented in NHPs with laser-induced ExGl. A principal limitation of our investigation is the lack of knowledge as to the mechanism of action or class of the topical agents previously administered to these NHPs, as well as the need for sporadic to frequent treatment to manage excessively high IOP in 4 eyes with ExGl in Set B. There are known structural effects of prostaglandin analogs (PGAs) and beta blockers on the TM^37–41^. In NHPs, sustained, long-term administration of PGAs and beta blockers have been shown to structurally alter the TM *in vivo*, with changes ranging from loss of TM and partial collapse of SC to complete rarefaction, respectively^40,42^. However, we believe any confounding effect of this treatment on our results would be mitigated by the short duration and intermittent nature of treatment in 3 of the 4 animals. Furthermore, the decrease in the elastic modulus in these 4 eyes suggested that tissue softening occurred to the same degree as 4 eyes not receiving any additional treatment with the aforementioned drugs during the year prior to euthanasia.

While chronic administration of PGAs and beta blockers may structurally alter TM, the mechanism of action of these drug classes does not specifically target TM to reduce IOP. Classes like the rho kinase (ROCK) inhibitors, however, specifically target the TM to increase conventional aqueous outflow and lower IOP by inducing rapid, but reversible, structural relaxation of associated smooth muscle^43–47^. Therefore, similar effects on the TM following administration of such agents to these animals must be considered when interpreting our results. Although we were masked to the class and mechanism of the test articles administered to these monkeys, we believe the study designs and nature of our findings also mitigate this possible confounding factor. Firstly, the vast majority of studies conducted using the animals presented here involved only brief, single-dose instillation of a test article, and none specified administration of any test article for greater than 3 days duration. The brevity of these studies were designed to minimize the possible effect of daily IOP fluctuation which can be a prominent limitation of this model.^48^ In this study, we report prominent morphological and ultrastructural changes in the ExGl eyes (i.e. decreased TM thickness, decreased giant vacuole formation). Such changes have not been reported in the past^49^, although more recently, Netarsudil, an inhibitor of Rho kinase/norepinephrine transporter, was reported to significantly increase the size of giant vacuoles in the Schlemm’s canal of non-glaucomatous eyes perfused *ex vivo* without altering its density^50^. Whether Netarsudil or other drug class have different effects on TM morphology/structure in glaucomatous vs non-glaucomatous models remains to be seen. In addition, even in the two animals with only a small difference in RNFL thickness between the ExGl eye and the control, the elastic moduli of the TM were consistent with the values of the other monkeys measured despite a marked difference in washout period prior to euthanasia in these two (2.7 months vs 26.9 months).

The data presented here suggest that a softer TM promotes increased outflow, provided by the capacity for unlasered primate TM cells in normal primate eyes to compensate for increased IOP and reduced overall outflow from the eye by altering the composition and subsequent mechanical properties of the matrix in the JCT region. *It is also noteworthy that, regardless of RNFL thickness, the elastic modulus in all eyes with ExGl were similarly softened, suggesting that the unlasered TM had reached a lower limit for compositional or organizational remodeling*. However, the time taken to achieve such softening could not be determined from the specimens available for investigation. To our knowledge, this is the first demonstration of tissue softening that highly suggests dynamic compensation of the normal NHP TM under supraphysiological load. Importantly, these data suggest that in the normal animal compensatory mechanisms exist that result in a softening of the meshwork in the face of chronically elevated IOP. The fact that glaucomatous humans exhibit a markedly increased stiffness (20-fold) suggests that individuals with POAG have a disruption of this compensatory mechanism. We acknowledge that the exact molecular mechanism(s) responsible for this compensation in the normal primate eye at this time remain unidentified. We also acknowledge that these tissue changes were observed in samples representing only a small portion (<10%) of the total TM, and that samples providing a temporal context of these TM changes, both immediately following laser treatment(s) and with respect to establishment of ocular hypertension would provide valuable insight. These data are beyond the scope of the current study, but we believe that the presence of a compensatory remodeling mechanism to explain our biomechanical results is well-supported by accompanying proteomic, morphological, and ultrastructural data.

Proteomic analysis of TM provides further insight into possible mechanisms of the tissue softening observed at the JCT in these samples. In eyes with ExGl, multiple matrix components were down-regulated including collagens and other structural matrix proteins. Recent studies using knockout mouse models lacking expression of MMP-9 have demonstrated spontaneous increased aqueous outflow resistance and ocular hypertension, in association with deposition of aberrant collagen in the TM^51,52^. Multiple enzymes related to cellular metabolism were also down-regulated and this may be a result of reduced cell numbers in the TM, as previously observed in glaucomatous patients^53–55^. Rapid alterations in TM mechanics are feasible as recently demonstrated by our group using a steroid-treated rabbit model^56^. Once softened to baseline level, only some remodeling of the ECM might be necessary and could signal TM cells to modify their metabolism since renewal of the ECM might be severely curtailed subsequently. These data would be strengthened by comparison to proteomic data from lasered TM tissue in ExGl eyes. These data, however, are not available at this time, and limit interpretation of our proteomic findings.

The morphological analysis presented here clearly demonstrates thinning of the unlasered regions of the TM in eyes with ExGl, with the SC collapsed and with substantially decreased giant vacuole formation. Concurrently, proteomic analysis demonstrated a clear downregulation of metabolic, structural, and matricellular proteins accompanied by a reduction in the elastic modulus emphasizing a dysfunctional meshwork. The findings from this analysis also suggest that compensatory softening and increased aqueous flow may correspond to ultrastructural abnormalities such as disruption of cellular processes, local cellular tight junctions and/or adhesion to the extracellular matrix, and loss of giant vacuole formation. Previous studies have reported increased ability for the expansion of giant vacuoles with increased IOP in normal eyes^7,57–60^, and an inhibited ability for both pore and giant vacuole formation in POAG^61–63^. Data from this study show decreased vacuole formation of inner wall endothelial cells of SC in normal cynomolgus eyes with long term elevation of IOP as a result of major alterations in the JCT. When taken in context with our findings, these reports suggest that up-regulation of vacuole formation is lost with time in the face of elevated IOP, and may not reflect a primary molecular dysregulation in matrix dynamics.

Furthermore, studies have shown that aqueous humor outflow from the eye is segmental, being high in some regions of the TM, and low in others^64,65^. Therefore, novel therapeutic agents that specifically target the TM should ideally aim to upregulate aqueous egress in lower-flow areas. Interestingly, the TM moduli for all 8 monkeys with ExGl in this study were approximately the same, regardless of the difference in IOP values between eyes with ExGl and controls, suggesting that the observed changes may represent an outer limit of compensatory softening. The discovery that compensatory matrix softening is intrinsic to this ExGl model suggests strong limitations as to its predictive value in drug discovery and development programs. Thus, potentially viable therapeutic agents could be withdrawn because of apparent lack of efficacy (i.e. false negative). If a drug’s mechanism of action is to enhance aqueous outflow and reduce IOP by altering the extracellular matrix, this model does not represent an ideal test system.

In conclusion, these data provide important insights into anterior and posterior segment changes in the NHP model of laser-induced ExGl. Despite chronic elevation in IOP, considerable RNFL thinning may not be observed in some monkeys, and may not correlate with all electrodiagnostic parameters. These results suggest that such discrepancies may be due to underlying genetic/epi-genetic mechanisms that are yet to be identified in future studies. This is particularly important considering that not all human POAG patients observe direct correlation between IOP and RNFL thinning. Despite structural and functional variation at the level of the retina *in vivo*, *ex vivo* analyses demonstrate more consistent findings. Ultrastructurally, the unlasered TM is thinned and relatively acellular with reduced giant vacuole counts along the inner wall of SC. The TM at the JCT is invariably and maximally softened in eyes with ExGl. Correspondingly, proteomic analysis demonstrates consistent downregulation of numerous structural proteins and metabolic enzymes. Collectively, these findings suggest dynamic compensation within the TM in normal animals in response to elevated IOP, a critical response that may be lacking in human patients with POAG, and underscore the need for further investigation of structural changes to the aqueous outflow pathway in this model. Importantly, despite *in vivo* findings that support use of this model in investigations of neuroprotection, the presence of compensatory alterations in the aqueous outflow pathway in unlasered regions limit the utility of this model in evaluation of therapies that target the TM.

## Materials and Methods

### Animals

Thirty-two eyes of 16 adult female cynomolgus macaques (*Macaca fascicularis*) were analyzed in the course of this study. All experimental methods and procedures were approved by the Animal Care and Use Committee at Covance Laboratories, Inc. (Madison, WI), and were performed consistent with the ARVO Statement for the Use of Animals in Ophthalmic and Vision Research. At least 40 months prior to euthanasia and globe collection, all animals had undergone unilateral laser trabeculoplasty (right eye [OD] only) to induce ExGl. A total of 3 diode laser treatments were used to ablate the majority (approximately 11 clock-hours) of the TM of all right eyes to induce unilateral ExGl. Thereafter, all animals had been used in preclinical investigations evaluating the hypotensive efficacy of topical test substances. In those investigations, all personnel involved were masked to the identity of the test articles. The vast majority of these investigations involved instillation of a single topical dose of a test article and measurement of IOP for a 24 hour period, followed thereafter by at least 2 weeks of washout period without any treatment. No studies involved administration of any test article for greater than 3 days duration. Prior to euthanasia, all animals had undergone washout periods (median 22.4 months; range 2.0–37.0 months) without any topical test articles prior to euthanasia. During the year prior to euthanasia, four of the animals in Set B (Numbers 3–6) required variable treatment with a topical beta blocker or prostaglandin analog (PGA) to manage excessively elevated IOP ( ≥ 40 mmHg) and ensure animal welfare. In three of these animals this treatment was sporadic, last performed 51 days prior to euthanasia in two, and 15 days prior to euthanasia in one. One animal (Number 6) required more frequent treatment, even until the day prior to euthanasia. Inclusion criteria (age, duration between onset of glaucoma and euthanasia, and washout period) for all animals, designated as Sets A-C, are presented in Table 3. All *in vivo* and *ex vivo* measurements and procedures performed for these animals are summarized in Table 1. Whole globes from Set A animals were submitted for confocal, light, and transmission electron microscopy (TEM); corneoscleral rims from Set B and C animals were submitted for proteomic analysis and/or atomic force microscopy (AFM).

**Table 3:** Inclusion criteria for all animals with unilateral (right eye [OD] only) experimental glaucoma (ExGl).

	Animal ID	Age (months)*	Duration Between Onset of ExGl and Euthanasia (months)	Washout Period Prior to Euthanasia (months)
Set A** (N = 3)	21	116.9	75.4	2.3
	34	113.5	58.8	26.4
	32	109.5	58.8	2.0
Set B† (N = 6)	1	149.5	119.2	24.1
	2	153.5	115.6	21.2
	3	76.1	44.7	23.9
	4	101.7	56.9	37.0
	5	79.0	44.5	23.7
	6	86.8	40.1	27.3
Set C†† (N = 7)	28	98.8	63.6	2.7
	36	104.8	59.2	26.9
	19	115.5	75.8	2.7
	24	115.7	75.4	33.4
	10	170.9	137.7	3.9
	18	114.7	75.4	2.3
	20	115.5	75.4	3.9

^*^At date of euthanasia.

^**^Whole globes submitted for confocal, light, and transmission electron microscopy.

^†^Corneoscleral rims submitted for proteomic analysis and/or atomic force microscopy.

^††^Corneoscleral rims submitted for proteomic analysis only.

### Laser Trabeculoplasty Procedure and Establishment of Experimental Glaucoma (ExGl)

All laser trabeculoplasty procedures were performed by a board-certified veterinary ophthalmologist with extensive experience in comparative glaucoma research. Details of surgical procedures are provided in supplementary materials.

### *In Vivo* Optical Coherence Tomography and Electrodiagnostics

Spectral-domain optical coherence tomography (SD-OCT) scans of the fundus were carried out in both eyes of all animals using a Zeiss™ Cirrus HD-OCT 4000 (Cwarl Zeiss, Oberkochen, Germany) and/or Heidelberg™ Spectralis HRA + OCT (Heidelberg Engineering, Heidelberg, Germany) instrument prior to euthanasia. Evaluation of scans focused on the RNFL, retinal ganglion cell layer (RNGL), optic nerve head (ONH), and macula. Segmentation and determination of RNFL thickness (RNFLT) was performed using standard automated algorithms as well as manual and combined (EdgeSelect™) techniques^66^. **S**cotopic and photopic full-field ERGs (ffERG) to a series of flash strengths, photopic flash visual-evoked potentials (fVEP), pattern electroretinograms (PERG) and pattern reversal visual evoked potentials (PRVEP), in that order. Measurements were recorded under anesthesia in two sessions using a BigShot^™^ electrodiagnostic system (LKC Technologies™, Gaithersburg, MD). Details on the experimental conditions and animal subsets are provided in supplementary materials.

### Collection and Fixation of Globes

Following euthanasia, all eyes were immediately enucleated and all right eyes with ExGl were marked to facilitate identification of the unlasered area of the TM. For AFM and proteomics (Sets B and C), corneoscleral rims were dissected, placed in Optisol™ and refrigerated before shipping from Covance Laboratories to the University of California – Davis. For confocal and light microscopy and TEM (Set A), whole globes were similarly enucleated and refrigerated, each placed in a moist container, and shipped refrigerated to Boston University within 24 hours of euthanasia.

### Atomic Force Microscopy (AFM)

Within 24 h post-euthanasia, tissue samples were received at UC Davis for AFM. After receipt, the unlasered portion of TM from right eyes (OD) with ExGl (Sets B and C) was microdissected according to data from the surgeon’s records, and the corresponding marking designated at enucleation. The elastic modulus determined using AFM according to methods previously described for human TM^13,56,67^. A similar dissection was done for the normal left eyes (OS) and AFM performed using a portion of TM of approximately the same size as the samples from eyes with ExGl. All force vs. indentation curves were obtained using the MFP-3D BIO system (Asylum Research™, Santa Barbara, CA). Force curves (5 per location) were obtained from 5 random locations along the length of the tissue.

### Proteomics

Within 24 h post-euthansia, tissue samples were received at UC Davis for proteomic analysis. Unlasered TM tissue was microdissected from ExGl eyes and controls using the technique described above. Isolated tissue samples were immediately digested in RIPA buffer, homogenized, and stored at −80 °C until analysis. Samples were precipitated and analyzed as described previously^56,68^. Mass spectra were collected in a data-dependent mode.

### Confocal, Light Microscopy (LM) and Transmission Electron Microscopy (TEM) Morphology

Immediately following enucleation, both eyes from Set A animals were refrigerated and shipped to Boston University for ocular perfusion, confocal, light microscopy (LM), and TEM of TM from untreated eyes and the non-lasered regions of eyes with ExGl. The last measurement of IOPs on the day of sacrifice were used for the analysis and to set the subsequent perfusion pressure.

#### *Ex vivo* ocular perfusion

Enucleated eyes were perfused with Dulbecco’s phosphate-buffered saline containing 5.5 mM D-glucose (GPBS) with the perfusion pressure set at the pre-mortem intraocular pressure minus 7 mmHg (episcleral venous pressure) for 15–30 minutes. This time was set based on previous reports for establishing a stable outflow facility^8^. A previously established two-color fluorescent tracer method^69^ was used to label the aqueous outflow patterns (green or red tracers; 500 or 200 nm; 0.002%). The anterior chamber contents were exchanged prior to perfusion with the same perfusates (tracers, GPBS, and fixative). All eyes were perfused with a fixed volume. Eyes were perfusion-fixed with modified Karnovsky’s fixative and immersed-fixed overnight with the same fixative before further processing.

#### Fluorescent tracer distribution in the TM and TM thickness by confocal microscopy

The eyes were hemisected along the equator, and the vitreous humor, ciliary body and lens were removed. Tracer distribution from the TM sides of the anterior chamber was imaged by a global imaging technique^70^ using a 300mm lens on a 4000MP VersaDoc™ imaging system (Bio-Rad Laboratories, Hercules, CA). The tracer distributions from the TM served as guides for later dissection of the hypertensive eyes correlating to non-lasered regions where tracers were observed, and lasered regions where tracers were not observed (Fig. S4 **)**. The anterior segments of the eyes were further divided into smaller wedges. For normal control eyes, tissue regions similar to the non-lasered regions of hypertensive eyes were used to analyze the TM thickness with confocal microscopy (Carl Zeiss 510, Axiover™ 200 M Laser Scanning Microscope, Carl Zeiss) and the Schlemm’s canal/juxtacanalicular tissue (SC/JCT) regions with electron microscopy. Each confocal image was measured at two to three different locations for TM thickness (the length from the innermost uveoscleral beam to the inner wall endothelium of SC), and the average thickness of TM in high-tracer regions of the ExGl and normal control eyes were then calculated and analyzed. The average fluorescence intensity was measured using ImageJ (v1.46; National Institutes of Health, Bethesda, MD, USA) and compared between ExGl and normal control eyes. For each image, a ~10 µm wide rectangular strip was drawn along the inner wall of SC. Average intensity per pixel was measured in the area in 8-bit images; 0 was the minimum and 255 was the maximum value of fluorescence intensity.

#### Giant vacuoles density and ultrastructure of the inner wall of Schlemm’s canal and juxtacanalicular tissue

After confocal imaging, semi-thin frontal tissue sections were fixed, cut, and stained with 1% Toluidine Blue (Fisher Scientific, Pittsburgh, PA). Light micrographs along the inner wall of the SC were taken using the Olympus™ FSX100 imaging system with a 40X objective. The height of SC (the distance between the inner and outer walls of SC) was measured and compared between normal control and ExGl eyes. A total of 6–12 measurements along SC were made to obtain the average SC height for each eye. The number of giant vacuoles was counted along the inner wall of SC where the SC was open. Ultra-thin sections were cut and the ultrastructure of the inner wall of SC and JCT regions were examined with a transmission electron microscope (JOEL™ JEM-1011, Tokyo, Japan). The percentage of SC length missing inner wall cells was then measured from the electron micrographs.

### Statistical analysis

Differences in ERG parameters were evaluated by paired-sample t-tests. Correlation with SD-OCT RNFL thickness was assessed with non-parametric Spearman’s rho coefficient. For AFM measurements, elastic modulus was represented as either box plots or bar charts (mean ± standard error in mean) from both eyes. Statistical significance was determined by performing unpaired Student’s t-test between the two groups for each animal (*p < 0.05, **p < 0.01, ***p < 0.001). To ascertain quantitative differences in biochemical composition of the TM derived from the lasered and unlasered eyes, nano-scale liquid chromatography tandem-mass spectrometry was performed. Proteomics data were analyzed using the built-in features of Scaffold viewer (Proteome Software Inc., OR). Normalized total spectral counts from the TM samples were compared for fold-change between the OS and OD eyes. Fisher’s exact test was performed to account for relative abundance in proteins between the groups. For morphological analysis, unpaired two-tailed Student’s t-test was used for statistical analysis; results are shown as mean ± standard deviation (SD).

## Electronic supplementary material

Supplementary Information
