# Supplementary material for: Biomechanical, ultrastructural, and electrophysiological characterization of the non-human primate experimental glaucoma model
Source: Sci Rep. 2017 Oct 30;7:14329. doi: 10.1038/s41598-017-14720-2 (PMC5662689; doi:10.1038/s41598-017-14720-2)
Supplement: Supplementary file 1 — Supplementary Information [file 41598_2017_14720_MOESM1_ESM.pdf]

Biomechanical, ultrastructural, and electrophysiological characterization of the non-human primate experimental glaucoma model

Authors: VijayKrishna Raghunathan[1,2,3#], J. Seth Eaton[1,2,#], Brian J. Christian[4], Joshua T. Morgan[1], James N. Ver Hoeve[2,5], Chen-Yuan Charlie Yang[6,7], Haiyan Gong[6,7], Carol A. Rasmussen[2,5], Paul E. Miller[2,8], Paul Russell[1,2], *T. Michael Nork[2,5], *Christopher J. Murphy[1,2,9]

Affiliations: [1]Department of Surgical and Radiological Sciences, School of Veterinary Medicine, University of California – Davis, Davis, California, 95616
[2]Ocular Services On Demand (OSOD), Madison, Wisconsin 53719
[3]The Ocular Surface Institute, Department of Basic Sciences, College of Optometry, University of Houston, Houston, Texas, 77204
[4]Covance Laboratories, Inc., Madison, Wisconsin 53704
[5]Department of Ophthalmology and Visual Sciences, School of Medicine and Public Health, University of Wisconsin – Madison, Madison, Wisconsin 53792
[6]Department of Anatomy and Neurobiology, School of Medicine, Boston University, Boston, Massachusetts 02118
[7]Department of Ophthalmology, School of Medicine, Boston University, Boston, Massachusetts 02118
[8]Department of Surgical Sciences, School of Veterinary Medicine, University of Wisconsin – Madison, Madison, Wisconsin 53706
[9]Department of Ophthalmology & Vision Science, School of Medicine, University of California – Davis, Sacramento, California 95817

[#] contributed equally to the work presented here and should therefore be regarded as equivalent authors

<u>Co-Corresponding Authors</u>:

T. Michael Nork, MD, MS
Department of Ophthalmology and Visual Sciences
University of Wisconsin Medical School
600 Highland Ave, K6/456
Madison, WI 53792-4673
tmnork@wisc.edu

Christopher J. Murphy, DVM, PhD, DACVO
School of Veterinary Medicine
University of California – Davis
1 Shields Avenue
1423 Tupper Hall
Davis, CA 95616
cjmurphy@ucdavis.edu

SUPPLEMENTAL FIGURES

Fig S1. Scatterplots (see **Fig. 2** for details) for a- and b-waves from 2.5, 4.0 and 14.0 cd-s m-2 flash intensities recorded under light adapted (top row) and dark adapted (bottom row) conditions. Although there are significant differences between control (blue triangle) and ExGl (red circles) eyes, there is no consistent evidence of a correlation between these measures and loss of nerve fiber layer thickness. Similar results were found for 30 Hz flicker (not shown).

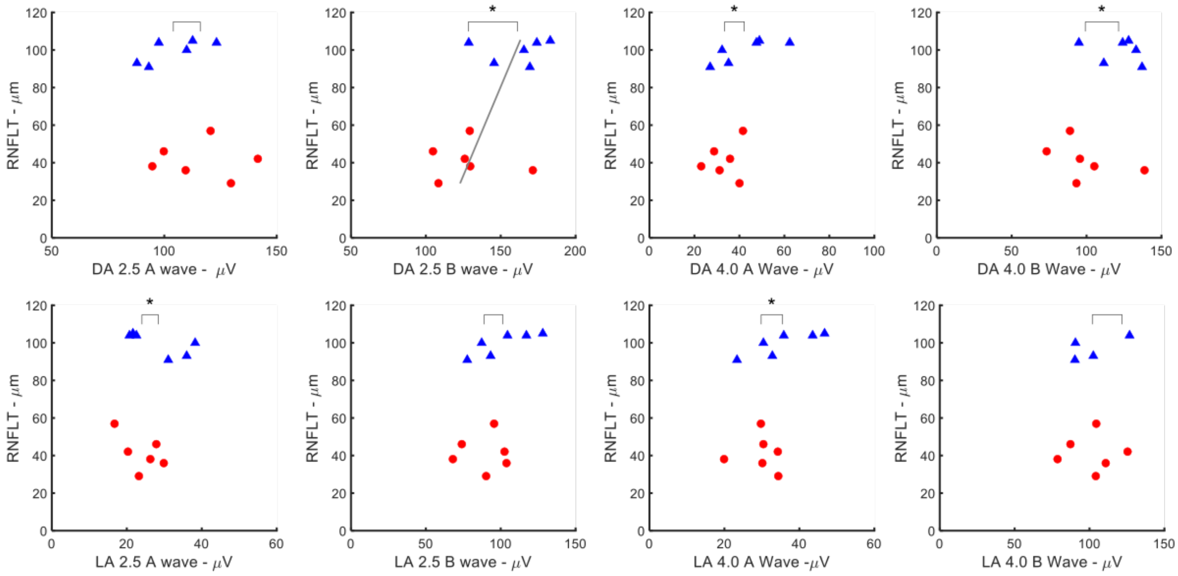

Fig S2. Histogram representing the elastic moduli of TM with respect to difference in intraocular pressure (IOP) at the time of euthanasia in individual animals between normal eyes (blue) and eyes with ExGl (red).

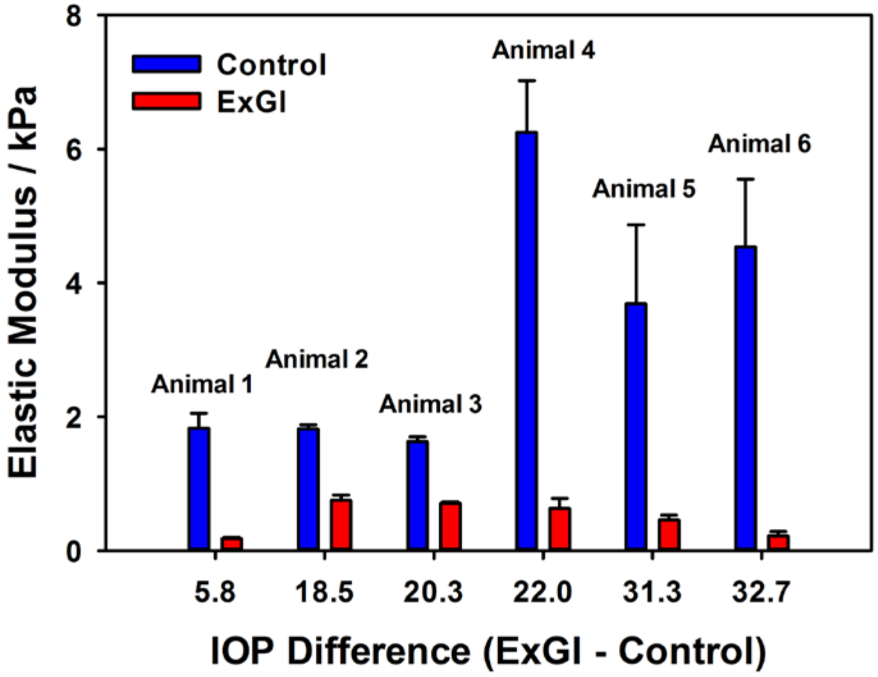

Fig S3. Principal component analysis plots: scree plot identifying the number of principal components that define the proportion of variance in data is illustrated. Plot of PC1 versus PC2 demonstrates data clustering for Control (OS) vs ExGl (OD) tissue samples.

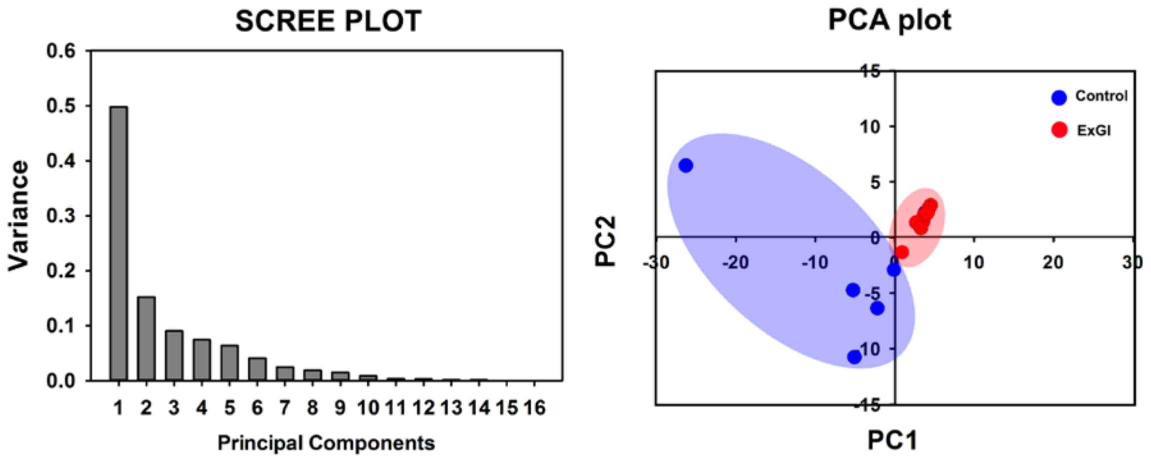

Fig S4. Global image of the fluorescent tracers in the trabecular meshwork (TM) in eyes of monkeys with EG (hypertensive) and fellow untreated controls (normal). The tracers' distribution in the TM (posterior view of the eye) from all three pairs of eyes is shown, which served as guides for later dissection of the hypertensive eyes to correspond non-lasered regions, where tracers were often observed and lasered regions where tracers were mostly not observed.

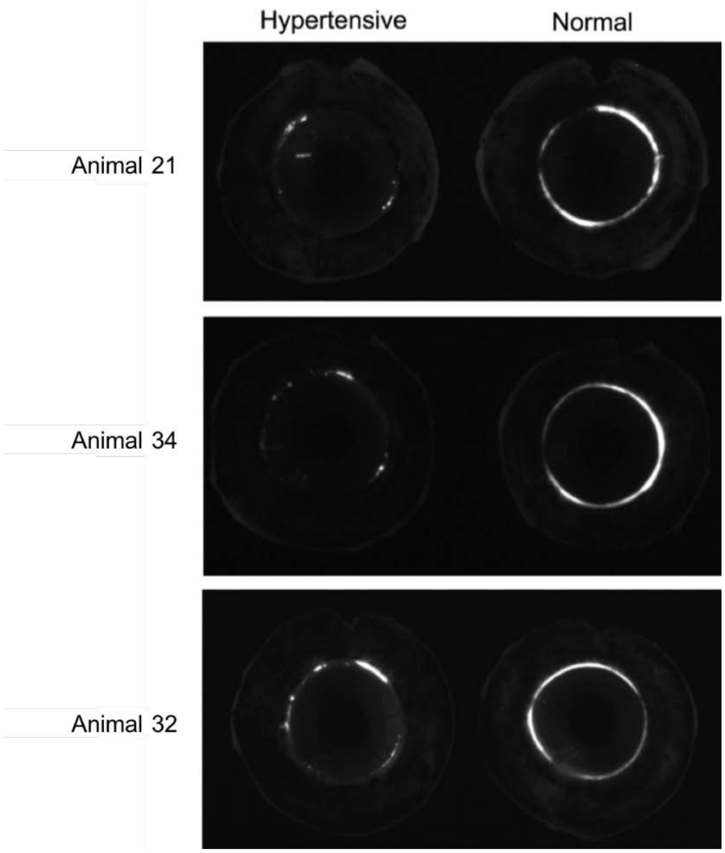

MATERIALS & METHODS

Animals

Thirty-two eyes of 16 adult female cynomolgus macaques (*Macaca fascicularis*) were analyzed in the course of this study. All experimental methods and procedures were approved by the Animal Care and Use Committee at Covance Laboratories, Inc. (Madison, WI), and were performed consistent with the ARVO Statement for the Use of Animals in Ophthalmic and Vision Research. At least 3 years prior to euthanasia and collection of globes, all animals had undergone unilateral laser trabeculoplasty (right eye [OD] only) to induce ExGl. Prior to euthanasia and inclusion in the present study, all animals had been used in preclinical investigations evaluating the hypotensive efficacy of topical test substances. The identities and properties of these test substances are proprietary and cannot be disclosed. To avoid the potentially confounding effect of day-to-day IOP fluctuations in this model[52], treatment periods with any substance were typically short in duration (<48 hours), or involved administration of only a single topical drop applied followed by a 2-week monitoring period. This also mitigated possibly confounding long-term effects on the TM. Furthermore, all animals had undergone washout periods without any topical medications prior to euthanasia. The inclusion criteria (age, duration between onset of glaucoma and euthanasia, and washout period) for all animals, designated as Sets A-C, are presented in **Table 1**. All *in vivo* and *ex vivo* measurements and procedures performed for these animals are summarized in **Table 2**. Whole globes from Set A animals were submitted for confocal, light, and transmission electron microscopy (TEM); corneoscleral rims from Set B and C animals were submitted for proteomic analysis and/or atomic force microscopy (AFM).

Laser Trabeculoplasty Procedure and Establishment of Experimental Glaucoma (ExGl)

All laser trabeculoplasty procedures were performed by a board-certified veterinary ophthalmologist with extensive experience in comparative glaucoma research. Animals were anesthetized with ketamine (10 mg/kg IM) and xylazine (0.5 mg/kg IM), or ketamine (10 mg/kg IM) and dexmedetomidine (0.025 mg/kg IM). In animals receiving dexmedetomidine, anesthesia was reversed with atipamezole (0.25 mg/kg IM). A 532 nm diode laser (Oculight™ GL Diode Laser, Iridex Corp., Mountain View, CA) and a Kaufman Single Mirror Laser Lens specifically designed for NHPs (Ocular Instruments, Inc.™, Bellevue, WA) were used to ablate the majority (approximately 11 clock-hours) of the trabecular meshwork (TM) of all right eyes to induce unilateral ExGl. The initial procedure treated the inferior 180° of the TM, using a contiguous series of laser burns with a spot size of 75 microns (the smallest size for this laser) at an initial power of 1 W for 0.5 seconds. Treatment was focused on the non-pigmented portion of the TM, as contiguous treatment in the often heavily pigmented TM of cynomolgus macaques can result in a cyclodialysis cleft and permanent hypotony. This did not occur in any eyes used in this study. The amount of applied laser energy was adjusted to result in whitening or bubble formation of the TM without inducing hemorrhage. The second procedure used an identical approach to treat the superior 180°, except for up to approximately 1 clock-hour of TM. The anatomical location of the unlasered TM was recorded by the surgeon. The last treatment was performed a minimum of 3 years before euthanasia. All contralateral left eyes (OS) served as untreated normotensive controls.

Slit-lamp biomicroscopy and indirect ophthalmoscopy were regularly performed following laser treatments, and all observations were quantified using a modified McDonald-Shadduck clinical scoring system [53]. In all animals, postoperative aqueous flare and cells were transiently observed but did not persist clinically. Topical atropine was administered postoperatively to control iridocyclospasm, but topical steroids were not administered as to encourage fibrosis of the laser-treated TM. Intraocular pressure (IOP) was periodically measured using a Model 30 Classic Pneumotonometer (Medtronic Solan™, Jacksonville, FL) after corneal anesthesia (**Table 2**). All animals were trained to accept IOP measurements without sedation. The only exception was the IOP measurement just prior to sacrifice during which the animals were lightly sedated with ketamine. In NHPs, however, there is evidence that ketamine alone does not lower IOP unless it is given multiple times over multiple consecutive days [54]. It is noteworthy that 4 animals whose globes were processed for AFM required periodic topical treatment with levobunolol to control excessively high IOP (> 55 mmHg) in the year prior to euthanasia. We do not, however, believe this to have significantly confounded our data.

Spectral-Domain Optical Coherence Tomography (SD-OCT)

Spectral-domain optical coherence tomography (SD-OCT) scans of the fundus were carried out in both eyes of all animals using a Heidelberg™ Spectralis HRA+OCT (Heidelberg Engineering, Heidelberg, Germany) instrument prior to euthanasia. Animals were anesthetized with ketamine (10 mg/kg IM), oxymorphone (0.15 mg/kg), and dexmedetomidine (0.025 mg/kg IM). Anesthesia was reversed with atipamezole (0.25 mg/kg) and naloxone (0.2 mg/kg). Set A animals (N=3) were scanned twice, once with both the Spectralis and a Zeiss™ Cirrus HD-OCT 4000 (Carl Zeiss, Oberkochen,

Germany) on the same day, and again 8 months later with the Spectralis. For all animals (N=16), the following were collected: macular scans (Heidelberg macular volume / Cirrus macular cube), high resolution line scans through the macula, high resolution line scans through the optic nerve, and retinal nerve fiber layer (RNFL) scans (Heidelberg glaucoma circle / Cirrus optic nerve cube). Evaluation of scans focused on the RNFL, retinal ganglion cell layer (RNGL), optic nerve head (ONH), and macula. Segmentation and determination of RNFL thickness (RNFLT) was performed using standard automated algorithms as well as manual and combined (EdgeSelect™) techniques [55].

Electrodiagnostics

All animals were anesthetized for electrodiagnostics. For ffERG and VEP, animals were anesthetized with ketamine (10 mg/kg IM) and dexmedetomidine (0.025 mg/kg IM); and anesthesia was reversed with atipamezole (0.25 mg/kg). For PERG, animals were anesthetized with ketamine (10 mg/kg IM), oxymorphone (0.15 mg/kg), and dexmedetomidine (0.025 mg/kg IM); and anesthesia was reversed with atipamezole (0.25 mg/kg) and naloxone (0.2 mg/kg).

In Set B animals (N=6), photopic and scotopic full-field ERGs (ffERG) to a series of flash strengths, photopic flash visual-evoked potentials (fVEP), pattern electroretinograms (PERG) and pattern reversal visual evoked potentials (PRVEP) were recorded in two sessions using a BigShot™ electrodiagnostic system (LKC Technologies™, Gaithersburg, MD).

Following a minimum of 2 h of dark adaptation, animals were routinely anesthetized and pupils were dilated with 1% tropicamide and 2.5% phenylephrine hydrochloride. Corneas were anesthetized with topical 0.5% proparacaine prior to application of ERG-

jet™ (Universo,™, Switzerland) contact lens electrodes and a conductive wetting solution. Reference electrodes were subdermal stainless steel needle electrodes inserted near the ipsilateral outer canthus of each eye. Visual evoked potentials were recorded from two active stainless steel subdermal electrodes situated approximately 1 cm superior to the occipital ridge and 1 cm lateral to the midline; VEP reference electrodes were situated adjacent to one another along the midline at the vertex. Dark-adapted ERGs were recorded, followed by 10 min of light adaptation, followed by the light-adapted series. Following full-field ERG testing, flash-evoked VEPs and PERGs and PRVEPs were recorded. For pattern stimulation recordings, animals were refracted for the viewing distance of 30 cm.

ERG and VEP waveforms were processed off-line and machine scored using software written in Matlab™ (Nattick, MA). Oscillatory potentials (OPs) were derived from the 2.5 cd-s m-2 flash conditions and bandpass filtered between 70 and 170 Hz. OPs and flash VEPs were quantified as the root-mean-square of the response from 10-160 msec post flash.

Collection and Fixation of Globes

Following euthanasia, all eyes were immediately enucleated and all right eyes with ExGl were marked to facilitate identification of the unlasered area of the TM. For AFM and proteomics (Sets B and C), corneoscleral rims were dissected, placed in Optisol™ and refrigerated before shipping from Covance Laboratories to the University of California – Davis. For confocal and light microscopy and TEM (Set A), whole globes were similarly enucleated and refrigerated, each placed in a moist container, and shipped refrigerated to Boston University within 24 hours of euthanasia.

Atomic Force Microscopy (AFM)

Within 24 h post-euthansia, tissue samples were received at UC Davis for AFM analysis. After receipt, the unlasered portion of TM from right eyes (OD) with ExGl (Sets B and C) was microdissected according to data from the surgeon's records, and the corresponding marking designated at enucleation. The elastic modulus determined using AFM according to methods previously described for human TM [13,42,56]. A similar dissection was done for the normal left eyes (OS) and AFM performed using a portion of TM of approximately the same size as the samples from eyes with ExGl. Briefly, all force vs. indentation curves were obtained, with the TM tissue placed in Hank's Balanced Salt Solution (HBSS), using the MFP-3D BIO system (Asylum Research™, Santa Barbara, CA). Force curves (5 per location) were obtained from 5 random locations along the length of the tissue. All force curves were obtained within 1 h of dissection to mitigate effects from any inadvertent degradation of tissues.

Proteomics

Within 24 h post-euthansia, tissue samples were received at UC Davis for proteomic analysis. Unlasered TM tissue was microdissected from ExGl eyes and controls using the technique described above. Isolated tissue samples were immediately digested in RIPA buffer, homogenized, and stored at -80°C until analysis. Samples were precipitated and analyzed as described previously [42,57]. Mass spectra were collected in a data-dependent mode. Peptide fragmentation was performed using higher-energy collision dissociation (HCD) with a normalized collision energy (NCE) value of 27. Unassigned charge states as well as +1 and ions >+5 were excluded from MS/MS fragmentation. Tandem mass spectra were extracted and charge states were deconvoluted and deisotoped. All MS/MS samples were analyzed using X! Tandem (The GPM, thegpm.org; version X! Tandem

Sledgehammer (2013.09.01.1)). X! Tandem was set up to search the Uniprot Macaca fascicularis (29,946 entries) plus an equal number of reverse sequences and 60 common non-human laboratory contaminant proteins, assuming the digestion was enzymatic using trypsin. X! Tandem was searched with a fragment ion mass tolerance of 20 PPM and a parent ion tolerance of 20 PPM. Carbamidomethyl of cysteine was specified in X! Tandem as a fixed modification. Glu->pyro-Glu of the N-terminus, ammonia-loss of the N-terminus, gln->pyro-Glu of the N-terminus, deamidated of asparagine and glutamine, oxidation of methionine and tryptophan, and dioxidation of methionine and tryptophan were specified in X! Tandem as variable modifications. Scaffold™ (version Scaffold_4.3.0, Proteome Software Inc., Portland, OR, USA) was used to validate MS/MS-based peptide and protein identifications. Peptide identifications were accepted if they could be established at a 99.0% probability by the Scaffold Local FDR algorithm; this corresponded to a 0.22% spectra decoy FDR and a 4% protein decoy FDR with 1 identified peptide per protein. Protein probabilities were assigned by the Protein Prophen algorithm 13. Proteins that contained similar peptides and could not be differentiated based on MS/MS analysis alone were grouped to satisfy the principles of parsimony. Proteins sharing significant peptide homology were grouped into clusters.

Confocal, Light Microscopy (LM) and Transmission Electron Microscopy (TEM) Morphology

Immediately following enucleation, both eyes from Set A animals were refrigerated and shipped to Boston University for ocular perfusion, confocal, light microscopy (LM), and TEM of TM from both untreated eyes, and the non-lasered regions of eyes with ExGl. The

last measurement of IOPs on the day of sacrifice were used for the analysis and to set the subsequent perfusion pressure.

Ex vivo ocular perfusion

Enucleated eyes were perfused with Dulbecco's phosphate-buffered saline containing 5.5 mM D-glucose (GPBS) with the perfusion pressure set at the pre-mortem intraocular pressure minus 7 mmHg (episcleral venous pressure) for 15-30 minutes. This time was set based on previous reports for establishing a stable outflow facility [8]. A previously established two-color fluorescent tracer method [58] was used to label the aqueous outflow patterns (green or red tracers; 500 or 200 nm; 0.002%). The anterior chamber contents were exchanged prior to perfusion with the same perfusates (tracers, GPBS, and fixative). All eyes were perfused with a fixed volume. Eyes were perfusion-fixed with modified Karnovsky's fixative and immersed-fixed overnight with the same fixative before further processing.

Fluorescent tracer distribution in the TM and TM thickness by confocal microscopy

The eyes were hemisected along the equator, and the vitreous humor, ciliary body and lens were removed. Tracer distribution from the TM sides of the anterior chamber was imaged by a global imaging technique [59] using a 300mm lens on a 4000MP VersaDoc™ imaging system (Bio-Rad Laboratories, Hercules, CA). The tracer distributions from the TM served as guides for later dissection of the hypertensive eyes correlating to non-lasered regions where tracers were observed, and lasered regions where tracers were not observed (**Fig. S4)**. The anterior segments of the eyes were further divided into smaller wedges. For normal control eyes, tissue regions similar to the non-lasered regions of hypertensive eyes were used to analyze the TM thickness with confocal microscopy (Carl Zeiss 510,

Axiover™ 200M Laser Scanning Microscope, Carl Zeiss) and the Schlemm's canal / juxtacanalicular tissue (SC/JCT) regions with electron microscopy. Each confocal image was measured at two to three different locations for TM thickness (the length from the innermost uveoscleral beam to the inner wall endothelium of SC), and the average thickness of TM in high-tracer regions of the hypertensive and normal control eyes were then calculated and analyzed.

Giant vacuoles density and ultrastructure of the inner wall of Schlemm's canal and juxtacanalicular tissue

After confocal imaging, frontal tissue sections were post-fixed in 2% osmium tetroxide, 1.5% potassium ferrocyanide in water for 2 hours, followed by *en bloc* staining with uranyl acetate, dehydrated in an ascending series of ethanol, infiltrated with propylene aside and embedded in Epon-Araldite. Semi-thin sections were cut and stained with 1% Toluidine Blue (Fisher Scientific, Pittsburgh, PA). Light micrographs along the inner wall of the SC were taken using the Olympus™ FSX100 imaging system with a 40X objective. The number of giant vacuoles was counted along the inner wall of SC where the SC was open. Ultra-thin sections were cut and the ultrastructure of the inner wall of SC and JCT regions were examined with a transmission electron microscope (JOEL™ JEM-1011, Tokyo, Japan). The percentage of SC length missing inner wall cells was then measured from the electron micrographs.

Statistical analysis

Differences in ERG parameters were evaluated by paired-sample t-tests. Correlation with SD-OCT RNFL thickness was assessed with non-parametric Spearman's rho coefficient. For AFM measurements, elastic modulus was represented as either box plots or bar charts

(mean ± standard error in mean) from both eyes. Statistical significance was determined by performing unpaired Student’s t-test between the two groups for each animal (*$p<0.05$, **$p<0.01$, ***$p<0.001$). To ascertain quantitative differences in biochemical composition of the TM derived from the lasered and unlasered eyes, nano-scale liquid chromatography tandem-mass spectrometry was performed. Proteomics data were analyzed using the built-in features of Scaffold viewer (Proteome Software Inc., OR). Normalized total spectral counts from the TM samples were compared for fold-change between the OS and OD eyes. Fisher’s exact test was performed to account for relative abundance in proteins between the groups. For morphological analysis, unpaired two-tailed Student's t-test was used for statistical analysis; results are shown as mean ± standard deviation (SD).
